# Acceleration of ageing via disturbing mTOR‐regulated proteostasis by a new ageing‐associated gene PC4

**DOI:** 10.1111/acel.13370

**Published:** 2021-05-06

**Authors:** Long Chen, Fengying Liao, Jie Wu, Ziwen Wang, Zhongyong Jiang, Chi Zhang, Peng Luo, Le Ma, Qiang Gong, Yang Wang, Qing Wang, Min Luo, Zeyu Yang, Shiqian Han, Chunmeng Shi

**Affiliations:** ^1^ Institute of Rocket Force Medicine State Key Laboratory of Trauma, Burns and Combined Injury Third Military Medical University Chongqing China; ^2^ Department of Cardiology Geriatric Cardiovascular Disease Research and Treatment Center 252 Hospital of PLA (82nd Group Army Hospital of PLA) Baoding China; ^3^ Department of Hematology Southwest Hospital Third Military Medical University Chongqing China; ^4^ Breast and Thyroid Surgical Department Chongqing General Hospital University of Chinese Academy of Sciences Chongqing China; ^5^ Institute of Tropical Medicine Third Military Medical University Chongqing China

**Keywords:** ageing, mTOR, PC4, protein synthesis, proteostasis

## Abstract

Research on ageing‐associated genes is important for investigating ageing and anti‐ageing strategies. Here, we firstly reported that the human positive cofactor 4 (PC4), a multifunctional and highly conserved nucleoprotein, is accumulated and activated during ageing and causes global accelerated ageing process by disrupting proteostasis. Mechanistically, PC4 interacts with Sin3‐HDAC complex and inhibits its deacetylated activity, leads to hyper‐acetylation of the histones at the promoters of mTOR‐related genes and causes mTOR signalling activation. Accordingly, mTOR activation causes excessive protein synthesis, resulting in impaired proteostasis and accelerated senescence. These results reveal a new biological function of PC4 in vivo, recognizes PC4 as a new ageing‐associated gene and provides a genetically engineered mouse model to simulate natural ageing. More importantly, our findings also indicate that PC4 is involved in histone acetylation and serves as a potential target to improve proteostasis and delay ageing.

## Graphical abstract

Proposed hypothesis for PC4 accelerating ageing process by activating mTOR signalling through promoting histone acetylation. PC4 is increased with age and inhibits the deacetylation activity of sin3a‐HDAC complexes, causing the transcriptional activation of the mTOR‐related genes and promoting the protein synthesis while the protein folding and the degradation of misfolding protein decreases with age, resulting in impaired proteostasis and cellular senescence.

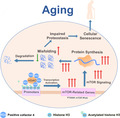

## 1 INTRODUCTION

Ageing, a complex, multifaceted process, has been recognized as a major factor for lifespan and age‐related diseases (Gorgoulis et al., 2019). There are some hypotheses trying to explain the causes of senescence involving DNA damage, shortening of telomeres, mitochondrial disorders and chromatin maintenance(Rai & Adams, 2013), but ageing remains an enigma (Gorgoulis et al., 2019). Some genes have been observed to change with age and might involve in the ageing process, which are called ageing‐associated genes. Studying the role and mechanisms of ageing‐associated genes is a common and popular strategy for anti‐ageing research.

Recent studies have shown a significant correlation between ageing and impaired proteostasis which is mainly regulated by protein synthesis, folding, trafficking and degradation (Denzel et al., 2014; Kaushik & Cuervo, 2015). To avoid the considerable risk of misfolding and aggregation and maintain proteostasis, proteins are ensured to fold, assemble and degrade efficiently (Alexander et al., 2019). Unfortunately, the function of the chaperones and the two proteolytic systems, the ubiquitin–proteasome and the lysosome–autophagy systems, which are responsible for protein folding and degradation, both are observed to decrease with age (Kaushik & Cuervo, 2015), causing the accumulation of the misfolded or aggregated protein resulting in unbalanced proteostasis and accelerated ageing (Tawo et al., 2017; Vila et al., 2019). Controlling protein synthesis is a readily adjustable target to maintain proteostasis and has been proved to delay ageing process (Takauji et al., 2016; Xie et al., 2019).

The recent studies showed that excessive protein synthesis caused changes of the cell size and cytoplasm dilution, which contributes to senescence (Neurohr et al., 2019; Polymenis & Kennedy, 2017). mTOR, a serine/threonine kinase in mammals, mainly responses to growth signals and promotes protein synthesis via some classical mTORC1 effectors such as S6K and 4E‐BP (Morita et al., 2015; Saxton & Sabatini, 2017), and protein synthesis is largely controlled by the mTOR pathway (Labbadia & Morimoto, 2015). As far as we know, mTOR is currently the only known pharmacological target which has been shown to delay ageing (Johnson et al., 2013). However, the side effects of currently available mTOR inhibitors have attracted wide attention (Zhang et al., 2019), which motivated the search for new regulatory strategy. Recently, histone modification has been proved to play an important role in the ageing process, and acetylation is observed to increase with age (Sen et al., 2019). However, the relationship of histone modification with mTOR signalling is vague.

The human positive cofactor 4 (PC4) or Sub1 in yeast has recently attracted great attention of researchers worldwide (F. Liao et al., 2020; Mondal et al., 2019; Sikder et al., 2019). PC4 has been considered as an evolutionarily conserved transcriptional co‐activator since the first identification (Ge & Roeder, 1994), which is involved in transcriptional activation (Calvo, 2018), oxidative stress (Yu et al., 2016) histone modification (Sikder et al., 2019), and maintaining genomic stability (Garavis & Calvo, 2017). In this study, for the first time, we find that PC4 is increased and becomes activated with age, and transgenic expression of PC4 disturbs mTOR‐regulated proteostasis and causes global accelerated ageing by promoting histone acetylation.

## 2 RESULTS

### 2.1 PC4 is increased and activated with age

**FIGURE 1:**
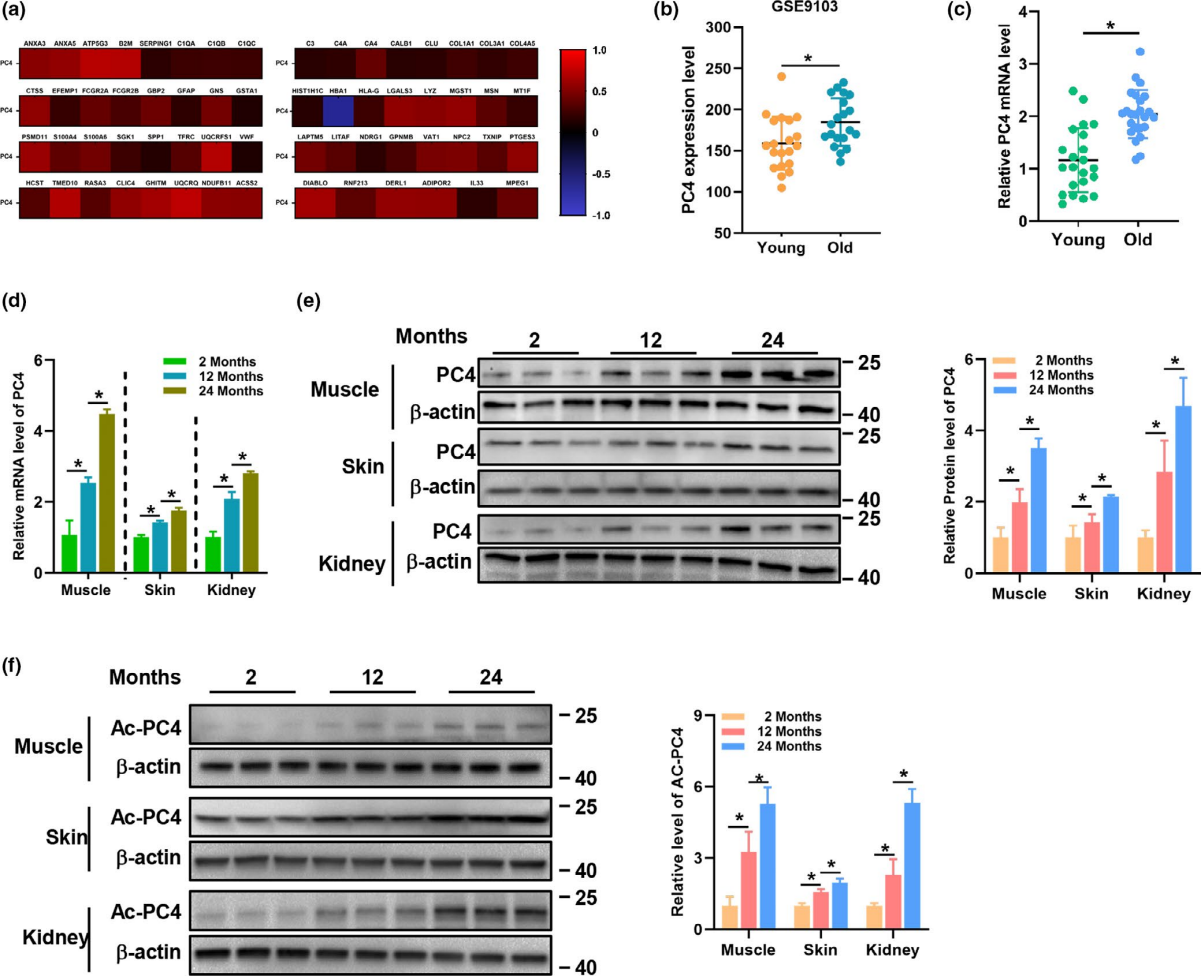
PC4 increased and activated during ageing. (a) Correlation analysis of PC4 and ageing‐associated genes using RNA‐seq data sets of in the human whole blood from GTEx. (b) PC4 mRNA level in muscle tissue of young (<45) and old (>45) healthy human participants from a data set (GSE9103), *n* = 20. (c) PC4 mRNA in peripheral blood of young and old healthy human volunteers. (d–f) PC4 mRNA (d), PC4 protein (e) and acetylated PC4 protein (f) level in muscle, skin and kidney from different month‐old mice, the animals of different ages were sacrificed at different days; then, the samples of mice were collected from independent groups, the experiments were performed until all the samples were collected. For (b, and c), and each symbol represents one participant, unpaired *t*‐test. For (d–f), *n* = 3 independent experiments. One‐way ANOVA (Dunnett), *p < 0.05

To study the possible relationship between PC4 and ageing, the correlation analysis between PC4 and 73 classical ageing‐associated genes (GenAge) in RNA‐seq data sets of normal tissues downloaded from the Genotype‐Tissue Expression (GTEx) was carried out. A positive correlation between PC4 and most of the ageing‐associated genes was observed in the whole blood (Figure 1a), skin (Figure S1a), kidney (Figure S1b) and the skeletal muscle (Figure S1c). To clarify the change of PC4 during ageing, we searched on GEO and collected a data set (GSE9103) containing the transcriptional signature from 20 young and 20 old healthy participants. Analysis of the data set showed that PC4 was increased in muscle tissue of old participants compared with the young participants (Figure 1b). For further verification, we collected peripheral blood samples from 46 healthy volunteers with different ages and analysed the mRNA expression level of PC4 in the young and the old samples. Our results also showed that higher expression of PC4 was observed in old volunteers (Figure 1c). In addition, the similar results were also found in multiple tissues including liver, skin and kidney from mice (Figure 1d,e). Previous studies have reported that post‐translational modifications (PTMs) including phosphorylation and acetylation determine the activity of PC4 (Dhanasekaran et al., 2016; Jonker et al., 2006). The phosphorylated form of PC4 generally was considered as inactive, while the acetylated form was considered as functional (Jonker et al., 2006). Interestingly, we found that the acetylated form of PC4 increased with age (Figure 1f). These results suggest that PC4 is increased and activated with age, indicating that PC4 might be involved in the ageing process.

### 2.2 Transgenic knock‐in of PC4 accelerates ageing in mice

**FIGURE 2:**
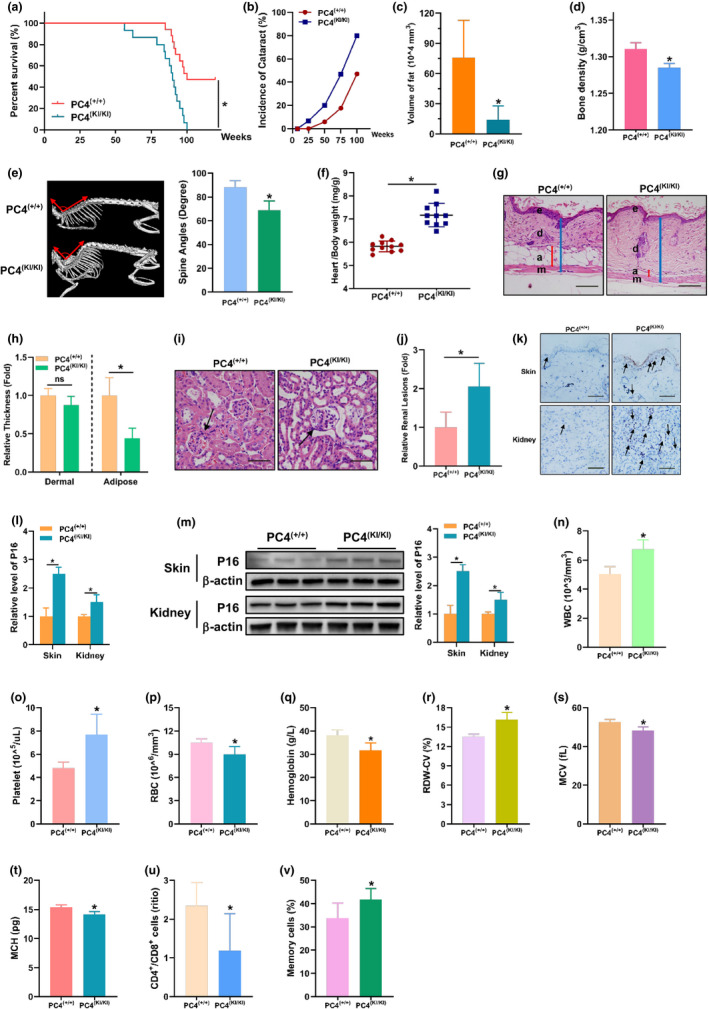
PC4 induced accelerated ageing phenotypes in multiple tissues of PC4 transgenic knock‐in mouse. (a) Life span analysis of PC4^(+/+)^ and PC4^(KI/KI)^ mouse. (b) Cataract incidence in PC4^(+/+)^ and PC4^(KI/KI)^ mice with age. (c–e) Analysis of fat level (c), bone density (d), and spine angles (e), heart‐to‐body weight ratio (f), skin dermal or adipose thickness (g and h), renal lesions (i and j), P16 level in skin and kidney (k–m), routine blood analysis (n–t), CD4+ to CD8+ T cell ratio (u) and percentage of memory T cells (v) in 18‐month‐old PC4^(+/+)^ and PC4^(KI/KI)^ mouse. For (a and b), *n* = 17 (PC4^(+/+)^), *n* = 15 (PC4^(+/+)^). For (c and d), *n* = 4. For (e, h, j and l), *n* = 5. For (f), each symbol represents one mouse. For (m), *n* = 3. For (n–t), *n* = 11 (PC4^(+/+)^), *n* = 10 (PC4^(+/+)^). For (u and v), *n* = 5 (PC4^(+/+)^), *n* = 6 (PC4^(+/+)^). For (a), Kaplan–Meier survival analysis. For (b–v), two‐tailed Student's *t*‐test. *p < 0.05

To better understand the role of PC4 in ageing, PC4 knock‐in (PC4^KI/KI^) mouse model was constructed since PC4 deficient mice were embryonically lethal (Liao et al., 2020). Overexpression of PC4 was confirmed at mRNA level (Figure S2a) and protein level (Figure S2b) in multiple tissues from homozygous PC4 knock‐in (PC4^KI/KI^) mice compared to wild‐type (PC4^+/+^) mice. Interestingly, no obvious abnormalities were observed in adult PC4^KI/KI^ mice (Video S1). However, overexpression of PC4 seemed to cause accelerated ageing phenotypes (Video S2). We found that PC4^KI/KI^ mice were shorter‐lived (Figure 2a) compared with PC4^+/+^ mice. In addition, typical markers during ageing such as opaque eyes (Figure 2b and Figure S2c), reduced fat content (Figure 2c) and bone density (Figure 2d) and kyphosis (Figure 2e) were observed earlier in PC4^KI/KI^ mice.

To further confirm the ageing phenotypes of PC4^KI/KI^ mice, mice were sacrificed and pathological analysis was performed. More heart weight gain is usually observed in ageing mice (Lessard‐Beaudoin et al., 2015). Our data showed that higher heart‐to‐body weight ratio was observed in PC4^KI/KI^ mice compared with PC4^+/+^ mice (Figure 2f). Subcutaneous adipose, an important ageing marker for skin (Tyner et al., 2002), reduced in PC4^KI/KI^ mice which was revealed through HE staining of skin (Figure 2g,h). In addition, more renal lesions including the decreased fractional mesangial area were also observed in PC4^KI/KI^ mice (Figure 2i,j). Similarly, more P16 positive cells, indicators of cellular senescence (Lessard‐Beaudoin et al., 2015), were also found in skin and kidney of PC4^KI/KI^ mice compared with PC4^+/+^ mice (Figure 2k,l), and this result was also verified through Western blot (Figure 2m).

In search of more evidence of accelerated ageing in PC4^KI/KI^ mice, the peripheral blood was analysed (Xie, Neff, et al., 2017). Our results showed that white blood cells (Figure 2n) and platelets (Figure 2o) increased, while red blood cells (Figure 2p) and haemoglobin (Figure 2q) decreased in PC4^KI/KI^ mice compared with PC4^+/+^ mice, which were consistent with reported ageing haematological parameters (Xie, Neff, et al., 2017). In addition, increased RBC distribution width (RDW; Figure S2r) values were observed which indicated stronger anisocytosis in PC4^KI/KI^ mice. Our data also showed that overexpression of PC4 caused increased mean corpuscular volume (MCV; Figure 2s) and mean corpuscular haemoglobin (MCH; Figure 2t) in mice. For the influences of age on T lymphocyte subsets, previous studies showed that the CD4^+^/CD8^+^ ratio usually reduced and accompanied by an increase in memory T cells (Xie, Zhang, et al., 2017). As expected, analysis of T lymphocyte subsets in peripheral blood showed that CD4^+^/CD8^+^ ratio (Figure S2u) decreased and memory T cells (Figure S2v) increased in PC4^KI/KI^ mice. These results showed that accelerated ageing was also observed in the blood and immune system of PC4^KI/KI^ mice.

In a word, the above results demonstrate that overexpression of PC4 causes global accelerated ageing phenotypes in mice.

### 2.3 PC4 accelerates senescence in vitro

**FIGURE 3:**
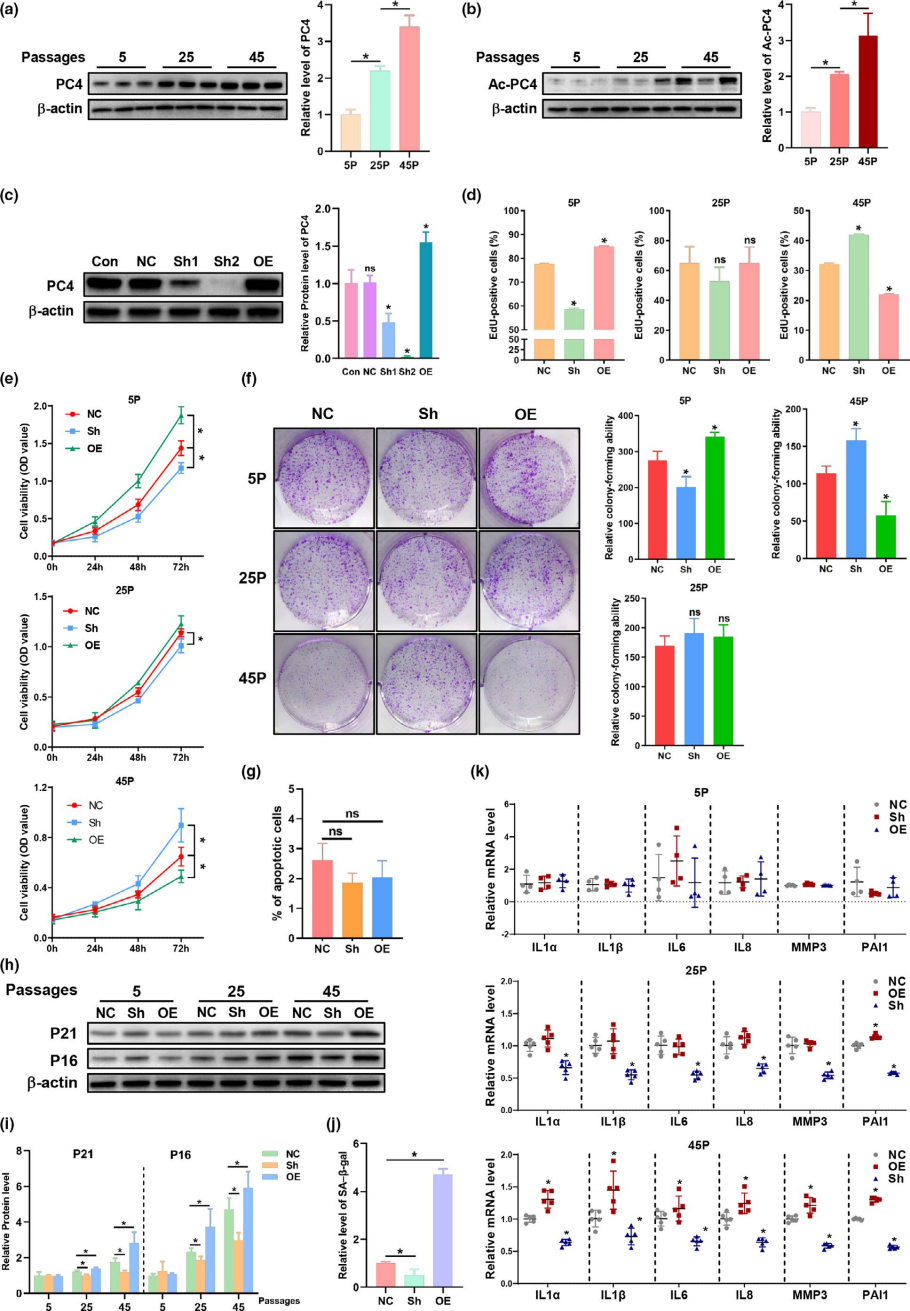
PC4 induced accelerated ageing phenotypes in PC4 transgenic knock‐in mouse. Total PC4 (a) and acetylated PC4 (b) level in 2BS cells at different passage. *n* = 3 *p < 0.05. (c) The PC4 protein level in stable PC4 knockdown and overexpression 2BS cell lines after lentivirus transfection with indicated shRNA. (d–g) EDU assay (d), cell viability detected by CCK‐8 assay (e), colony formation assay (f) and apoptosis ratio (g) in 2BS cells at indicated passage. (h–k) Senescence‐associated indicators including protein level of P16 and P21 (h and i), SA‐β‐gal (j) and senescence‐associated secretory phenotypes (SASP) in in 2BS cells at indicated passage. For (a–j), 5P, 25P and 45P were abbreviations of cells at 5, 25 or 45 passage. For (c–h), stable transfected 2BS cells with lentivirus carrying normal control (NC), knockdown (Sh) and overexpression (OE) shRNA of PC4. For (a–d), *n* = 3. For (e), *n* = 6. For (f–j), *n* = 3. For (k), *n* = 4. One‐way ANOVA, *p < 0.05

Then, the 2BS cell line, a human foetal lung diploid fibroblast cell line used to study replicative senescence in vitro which is usually considered as young until 30 passages and fully senescent above 55 passages (Dang et al., 2020), was used to further study the effects and mechanisms of PC4 on senescence in vitro. Consistent with the in vivo results, both total and acetylated form of PC4 increased with the passage number (Figure 3a,b). Then, we established PC4 negative control (NC), knockdown (PC4KD) and overexpression (PC4OE) stable 2BS cells (Figure 3c). Firstly, we detected the effects of PC4 on proliferation through EdU assay (Figure 3d), cell viability (Figure 3e) assay and colony formation assay (Figure 3f). Interestingly, we found that PC4OE cells proliferated faster while PC4KD cells proliferated more slowly both compared with PC4NC cells when 2BS cells were young (5P). However, the opposite phenotypes occurred when 2BS cells were in higher passages (45P), indicating that PC4OE cells seemed to reached senescence earlier while PC4KD delayed senescence process. In order to exclude the effect of apoptosis, we detected the apoptosis of the three types of 2BS cells. Our results showed that PC4 seemed to have no effect on apoptosis (Figure 3g). Then, we investigated senescence‐associated markers including senescence‐associated beta‐galactosidase (SA‐β‐gal), cell cycle‐related protein (P16 and P21) and senescence‐associated secretory phenotype (SASP). Our results showed that PC4OE cells gained more P21 and P16 while PC4KD gain less both compared with PC4NC at 45P of 2BS (Figure 3h,i). Furthermore, similar changes of SA‐β‐gal (Figure 3j) and SASP (Figure 3k) were observed. These results indicate that PC4 could accelerate replicative senescence of 2BS cells in vitro.

### 2.4 PC4 accelerates cellular senescence by decreasing proteostasis

**FIGURE 4:**
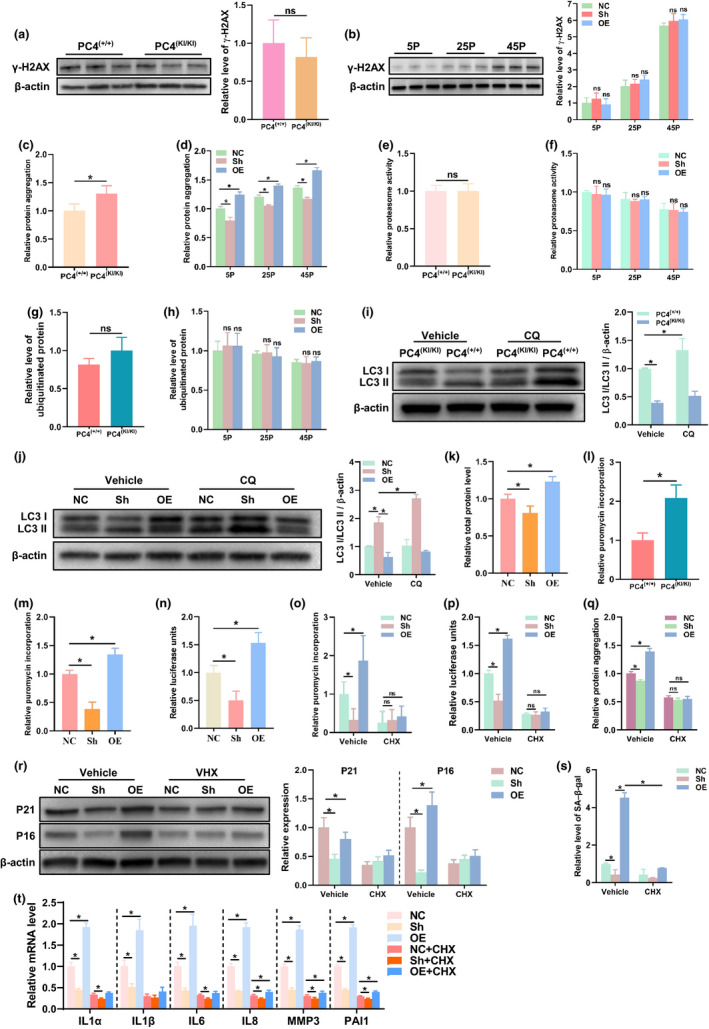
PC4 accelerates cellular senescence and promotes protein synthesis. (a and b) DNA damage level assessed by γ‐H2AX through Western blot in vivo (a) and in vitro (b), *n* = 3. (c and d) Proteostasis assessed by protein aggregation level in vivo (c) and in vitro (d), *n* = 5. (e–h) Statistical analysis of ubiquitin–proteasome degradation assessed by proteasome activity (e and f, *n* = 9) and ubiquitinated protein level (g and h, *n* = 3) in vivo and in vitro. (i and j) Autophagy level assessed by the ratio of LC3 II to β actin in vivo (i) and in vitro (j). CQ, Chloroquine, an autophagy inhibitor. *n* = 3. (k) Total protein concentration in whole cell lysate from same number of stable transfected 2BS cells by BCA protein assay, *n* = 6. (l–p) Statistical analysis of protein synthesis assessed by puromycin incorporation (l, m and o, *n* = 3) using Western blot and Luciferase reporter assay (n and p, *n* = 5). (q) Proteostasis assessed by protein aggregation level in 2BS cells, *n* = 5. (r–t) Senescence‐associated indicators including protein level of P16 and P21 (r, *n* = 3), SA‐β‐gal (s, *n* = 3) and SASP (t, *n* = 4) in in 2BS cells. For (a, c, e, g, i, l), 18‐month‐old PC4^(+/+)^ or PC4^(KI/KI)^ mice were used. For (i–t), 2BS cells at 45 passage were used. For (o–t), 0.1 μM of cycloheximide (CHX), an inhibitor of protein synthesis, was incubated with indicated 2BS cells for five consecutive passages. (a, c, e, g, l), two‐tailed Student's *t*‐test; (b, d, f, h, i, j, o, p, q, r, s, t), two‐way ANOVA; (k, m, n), one‐way ANOVA; *p < 0.05

DNA damage is an influential factor for senescence, and PC4 has been reported to be related to oxidative stress and genomic stability. However, our results showed that accelerated senescence induced by PC4 seemed to be independent on DNA damage level both in vivo (Figure 4a) and in vitro (Figure 4b). The aggregation of misfolded proteins also contributes to senescence via impaired proteostasis (Magalhaes et al., 2018). Then, we evaluated the protein aggregation level. Our results showed that PC4 seemed to increase protein aggregation in vivo (Figure 4c) and in vitro (Figure 4d). Then, we analysed the two important cellular processes including protein synthesis and protein degradation, and the latter is mainly controlled by ubiquitin–proteasome degradation pathway and lysosome–autophagy systems (Kaushik & Cuervo, 2015; Labbadia & Morimoto, 2015). We found that PC4 appeared to have no effect on the ubiquitin–proteasome degradation pathway (Figure 4e–h and Figure S3a,b), but PC4 inhibited the autophagy level (Figure 4i,j). Interestingly, we unintentionally found that PC4 caused higher total protein level in 2BS cells (Figure 4k). In addition, PC4 appeared to promote protein synthesis both in vivo and in vitro, which was proved by puromycin incorporation (Figure 4l,m and Figure S3c,d) and Luciferase reporter assay (Figure 4n). To verify whether accelerated senescence induced by PC4 was related to protein synthesis, cycloheximide (CHX), a specific inhibitor of protein synthesis, was used to incubate with 2BS cell for five consecutive passages. Interestingly, CHX inhibited the protein synthesis rate (Figure 4o,p and Figure S3e), decreased the protein aggregation (Figure 4q) and rescued the premature senescence‐related indicators in PC4OE 2BS cells (Figure 4r–t). These results suggest that PC4 accelerates senescence via impaired proteostasis by increasing protein synthesis and inhibiting autophagy.

### 2.5 PC4 disturbs mTOR‐regulated proteostasis

**FIGURE 5:**
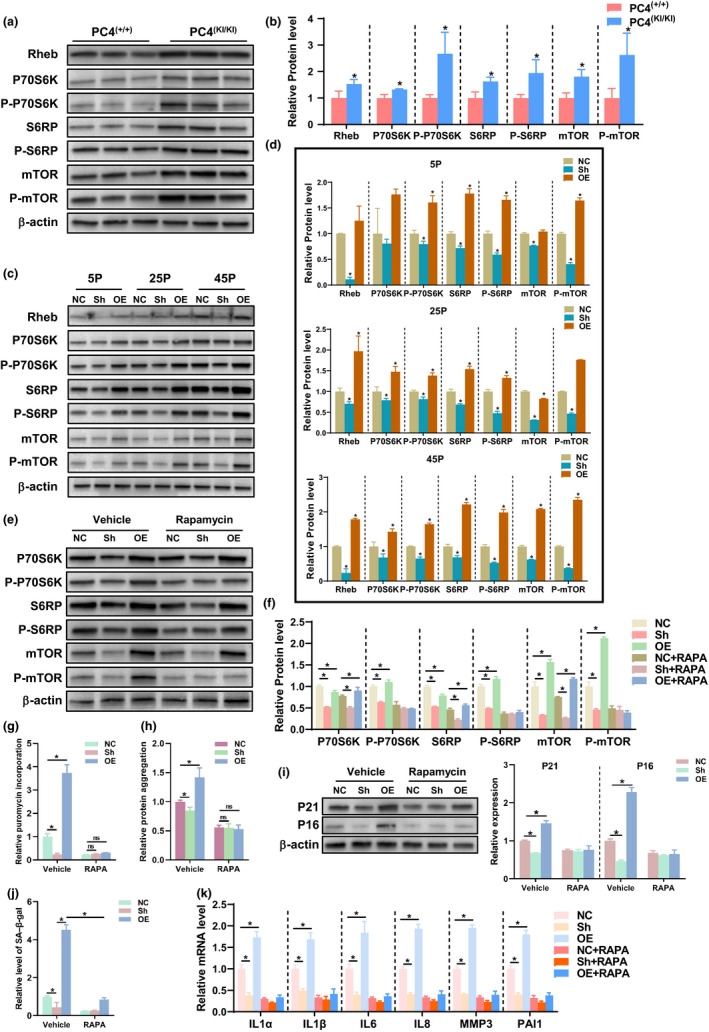
PC4 promoted protein synthesis and decreased proteostasis by activated mTOR signalling. (a–e) Results and statistical analysis of mTOR signalling‐related protein including Rheb, P70S6K, S6RP, mTOR and their phosphorylated forms were detected by Western blot in 18‐month‐old PC4^(+/+)^ or PC4^(KI/KI)^ mice (a and b) and in 2BS cells at indicated passage (c and d) *n* = 3. (e–k) Results of mTOR signalling‐related protein (e and f), protein synthesis assessed by puromycin incorporation (g), proteostasis assessed by protein aggregation (h) and senescence‐associated indicators including protein level of P16 and P21 (i, *n* = 3), SA‐β‐gal (j, *n* = 3) and SASP (k, *n* = 4) in 2BS cells after vehicle or RAPA treatment for five consecutive passages. Rapamycin (RAPA, 0.1 μM), a classical mTOR inhibitor. For (e, f, g, i and j), *n* = 3. For (h), *n* = 5. For (k), *n* = 4. For (b), two‐tailed Student's *t*‐test; For (d), one‐way ANOVA; For (f–k), two‐way ANOVA; *p < 0.05

mTOR signalling is one of the major regulatory pathways for protein synthesis and autophagy (Ravi et al., 2019). Then, we tested whether PC4 regulated mTOR signalling axis. Our data showed that PC4 activated mTOR signalling since the increased level of the phosphorylated mTOR and its downstream targets including p70S6K and S6 ribosomal protein (S6RP) were observed both in vivo (Figure 5a,b) and in vitro (Figure 5c,d). In addition, our results also showed that PC4 increased the protein level of Rheb which is one of the key activators for Mtor (Deng et al., 2019; Figure 5a–d). Then, we tested whether mTOR activation is required for accelerated senescence effect of PC4. Rapamycin (RAPA), a specific inhibitor of mTOR, reduced the phosphorylated forms of mTOR, p70S6K and S6RP (Figure 5e,f), slowed the protein synthesis rate (Figure 5g and Figure S4a), decreased the protein aggregation (Figure 5h) and rescued the accelerated senescence phenotypes in PC4OE 2BS cells (Figure 5i–k). In addition, RAPA treatment also decreased the ageing‐related protein level in PC4^KI/KI^ mice (Figure S4b). Interestingly, RAPA treatment seemed to have no effect on unphosphorylated forms of mTOR and its downstream targets (Figure 5e,f). These results indicate that PC4 accelerates senescence by disturbing mTOR‐regulated proteostasis.

### 2.6 PC4 transcriptionally activates the mTOR pathway by enhancing histone acetylation

**FIGURE 6:**
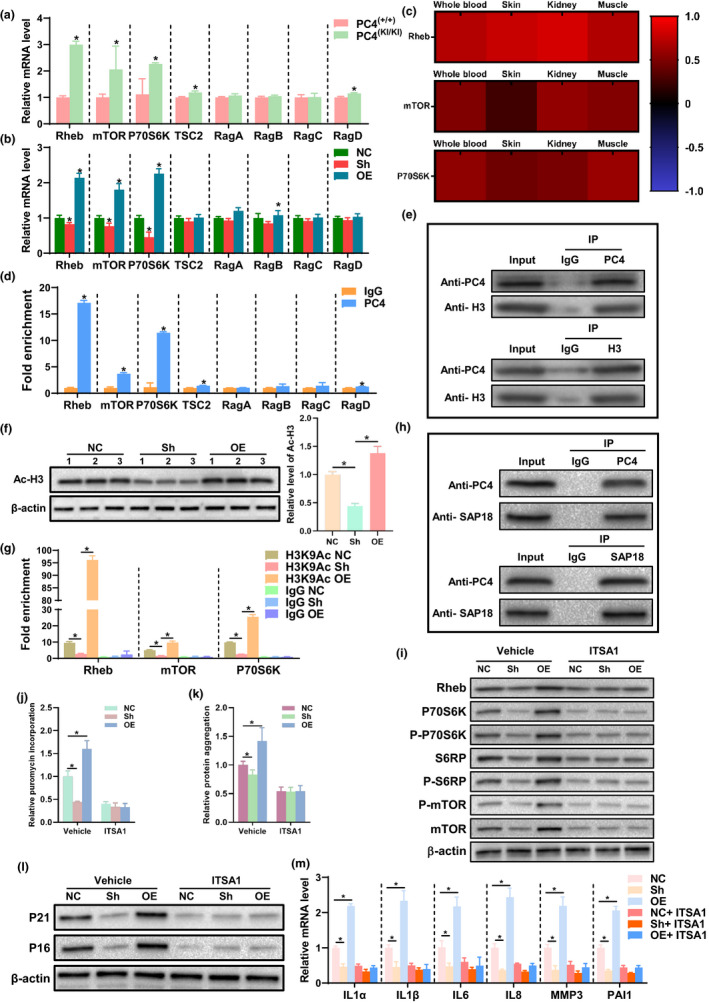
PC4 activated mTOR signalling by promoting histone H3 acetylation. (a and b) mRNA level of mTOR signalling‐related gene in 18‐month‐old PC4^(+/+)^ or PC4^(KI/KI)^ mice (a, *n *= 5) and in 2BS cells at 25 passage (b, *n* = 3). (c) Correlation analysis of PC4 and Rheb, mTOR, P70S6 K using RNA‐seq data sets of in multiple tissues including the whole blood, skin, kidney, and muscle from GTEx. (d) ChIP analysis of PC4 binding to the promoters of the mTOR‐related genes performed with a PC4‐specific antibody or IgG control antibody in 2BS cells. *n* = 3. (e) The interaction of PC4 and histone H3 was co‐immunoprecipated and detected by Western blotting. (f) Acetylated histone H3 level in 2BS cells, *n* = 3. (g) Enrichment of acetylated histone H3 (Ac‐H3) relative to IgG at the promoters of mTOR signalling‐related genes including Rheb, mTOR and P70S6K were analysed by CHIP assay using an Ac‐H3 specific antibody or IgG control antibody in 2BS cells, *n* = 3. (h) The interaction of PC4 and SAP18 was co‐immunoprecipated and detected by Western blotting. (i–m) The effects of vehicle or ITSA1 on mTOR‐related protein level (i, *n* = 3), protein synthesis (j, *n* = 3), proteostasis (k, *n* = 5), P21 and P16 level (l, *n* = 3) and SASP (m, *n* = 4). For (e and h), IgG antibody was used as negative control, and whole cell lysate (Input) was used as a positive control. ITSA1 (0.1 μM), a specific activator of histone‐deacetylated complex sin3a‐HDAC, was incubated with 2BS cells for five consecutive passages. For (a and d), two‐tailed Student's *t*‐test; For (b and f), one‐way ANOVA; For (g–m), two‐way ANOVA. *p < 0.05

To investigate the mechanism by which PC4 activates mTOR signalling, we firstly detected the transcription level of mTOR‐related upstream and downstream genes including Rheb, mTOR, p70S6K, TSC2, RagA, RagB, RagC and RagD. Our results showed that PC4 increased the mRNA level of these key genes for mTOR activation including Rheb, mTOR, p70S6K both in vivo (Figure 6a) and in vitro (Figure 6b). Then, we analysed the correlation between PC4 and the key regulators of mTOR in RNA‐seq data sets of normal tissues downloaded from the Genotype‐Tissue Expression. A positive correlation between PC4 and Rheb, mTOR and p70S6K was observed in the whole blood, skin, kidney and the skeletal muscle (Figure 6c). More importantly, the ChIP analysis showed significant enrichment of PC4 at the promoters of Rheb, mTOR, p70S6K (Figure 6d). These results indicate that PC4 might activate mTOR signalling at the transcription level.

As a transcriptional cofactor, PC4 has been reported to interact with some classical transcriptional factors (TFs; Liao et al., 2011). To study whether PC4 activated mTOR through binding to related TFs, co‐IP assays combined with mass spectrometry (MS) were performed using a Flag antibody in 2BS cells transfected with Flag‐tagged PC4 protein (Table S5). Then, the results were analysed and grouped into Functional Categories (UP KEYWORDS). Consistent with the above results, the Protein Biosynthesis was the most enriched, and the ribonucleoprotein, initiation factor and ribosomal protein were also enriched (Table S6). Histone modification is involved in transcriptional activation and ageing process. Interestingly, the results of the MS revealed that PC4 interacted with histone H3, and co‐IP assay in vitro also verified this result (Figure 6e). Histone H3 acetylation is associated with transcriptional activation, and PC4 has been reported to affect chromatin remodelling (Das et al., 2006). Interestingly, our data showed that PC4 caused a higher level of acetylated H3 in 2BS cells (Figure 6f). In addition, more enrichment of acetylated H3 at the promoters of Rheb, mTOR, p70S6K was also observed in 2BS cells (Figure 6g), suggesting PC4 might promote histone H3 acetylation. Then, we analysed the results of co‐IP assays combined with MS again. An unexpected discovery was that SAP18, an important component of Sin3‐HDAC complexes which was mainly responsible for histone deacetylation, was identified. Whether PC4 promoted histone H3 acetylation by interacting and inhibiting the deacetylation activity of Sin3‐HDAC complexes. To test this hypothesis, we verified the interaction of PC4 and SAP18 by co‐IP assay and Western blot. Our data showed confirmed the interaction of PC4 and SAP18 (Figure 6h), and more importantly, ITSA1, a classical activator of HDAC, inhibited the activation of mTOR signalling (Figure 6i), slowed the protein synthesis rate (Figure 6j), decreased the protein aggregation level (Figure 6k) and rescued the accelerated senescence in PC4OE 2BS cells (Figure S5a,b). These results suggest that PC4 binds to the promoters of mTOR‐related genes, interacts with SAP18, inhibits the deacetylated activity of Sin3‐HDAC complexes, caused hyper‐acetylated histone H3 and finally resulting in transcriptional activation of mTOR signalling.

## 3 DISCUSSION

Although there are few biological functions about PC4 in vivo(Swaminathan et al., 2016), our and others’ studies have demonstrated that PC4 is participated in transcriptional activation, oxidative stress, and genomic stability, cancer development and reprogramming of somatic cell in vitro, suggesting that PC4 is an important and multifunctional protein (Garavis & Calvo, 2017; Jo et al., 2016; Mortusewicz et al., 2016; Yu et al., 2016). Here, we firstly linked PC4 to the ageing process in vivo. We found that PC4 increased, activated during ageing and accelerated the global ageing process in mice. In our work, further our understanding of PC4 in vivo and suggested PC4 might be an ageing‐associated gene. In addition, artificial ageing animal models are helpful for investigating the precise mechanisms of the ageing. Although there are some helpful artificial mouse models for premature ageing syndromes (Gorgoulis et al., 2019), there are few mouse models for the natural ageing process. Here, our findings suggested that PC4^KI/KI^ mouse might be a potential accelerated natural ageing mouse model for anti‐ageing research.

Impaired proteostasis is related to the ageing process, and protein synthesis is a readily adjustable pharmacological or genetic target relative to other links for maintaining proteostasis (Ravi et al., 2019). In this study, we found that PC4 induced impaired proteostasis through promoting protein synthesis and resulting in accelerating cellular senescence. The mTOR signalling is the crucial pathway for regulating protein synthesis and the only known pharmacological target proved to delay ageing (Harrison et al., 2009). Interestingly, in this study, we found that PC4 activated mTOR signalling by promoting the transcription of key genes associated with mTOR signalling. Consistent with previous study (Sikder et al., 2019), our data also indicated that PC4 inhibited autophagy which was negatively regulated by mTOR signalling. Histone acetylation is related to open chromatin and promote transcription, and hyper‐acetylated histones are also observed during ageing (Sen et al., 2019). In addition, studies have indicated that mTOR activation with age might be associated with the change of histone modification (Wei et al., 2015), suggesting that histone modification might be a potential target for mTOR activation. Previous reports have suggested that PC4 might involve in histone modification (Sikder et al., 2019), although specific mechanisms are still unclear. Here, our results supported the conclusion that PC4 increases the acetylation of histone H3 (Zhao et al., 2018). Further, interestingly, we found that PC4 not only interacted with both H3 but also bound to SAP18 which is a part of Sin3‐HDAC complexes. In addition, PC4 also increased the enrichment of acetylated H3 at the promoters of P70S6K, Rheb and mTOR genes, suggesting PC4 might inhibit the deacetylation activity of Sin3‐HDAC complexes. More importantly, ITSA1, a classical HDAC activator, rescued the accelerated senescence induced by PC4 overexpression. Previous studies have suggested PC4 activates in response to multiple stressors such as ionizing radiation and oxidative injury (Mondal et al., 2019), and ageing is usually considered as a chronic but cumulative stressor (Hartl, 2016). Thus, we presented a possible assumption that PC4 increased and activated with age, inhibited the histone deacetylation at the promoters of mTOR activation‐related genes, caused progressive activation of mTOR with age and finally resulting in impaired proteostasis and accelerated ageing. Since the Sin3‐HDAC complex is an effective target for mTOR regulation and PC4 seemed to bound to the region related to mTOR activation, our findings suggested PC4 as a potential anti‐ageing target.

Overall, our findings suggest that PC4 is a possible ageing‐associated gene and involves in the ageing process, and PC4 knock‐in mouse might be a helpful feasible transgenic mouse model of accelerated natural ageing. Besides, PC4 could be a potential target to improve mTOR‐regulated proteostasis and delay ageing.

## 4 MATERIALS AND METHODS

### 4.1 Peripheral blood from human

Peripheral blood samples from healthy adult donors were obtained from the Blood Donation Center of Chengdu Military General Hospital, and this study was approved by the Ethics Committee of Army Medical University. Witnessed informed consent was obtained from each volunteer.

### 4.2 Animals

Mice were maintained on an ad libitum diet under specific pathogen‐free (SPF) conditions. All experiments were approved by the Animal Care and Use Committee of the AMU and conducted under the Guidelines for the Care and Use of Laboratory Animals of the AMU.

### 4.3 Generation of PC4 transgenic mice

Transgenic mice were generated as previously described (Liao et al., 2020). A 3′‐untranslated region endogenous mouse Sub1(PC4) track for a vector containing both the homologous arms (HAs) of the EF1α‐mouse Sub1(PC4) cDNA‐IRES‐eGFP‐PolyA knock‐in (KI) box is to ensure efficient homologous recombination (HR) (Liao et al. PC4 serves as a negative regulator of skin wound healing in mice. Burns Trauma 8, tkaa010 (2020). Figure 1a, as shown below). In the targeting vector, the positive selection marker (Neo) was flanked with LoxP sites. DTA was used for negative selection. The constitutive KI allele was obtained after Cre‐mediated recombination. We verified the fidelity of the targeted structures before direct Sanger sequencing of embryonic stem cells (ES cells). Transgenic mice were generated using the standard method (Cyagen Biosciences Inc.). After drug screening and polymerase chain reaction (PCR) confirmation, ES cell clones with correct HR were expanded and injected into blastocysts, which were then re‐implanted into pseudo‐pregnant females. Then, the mice with recombinant alleles (KI) were identified through PCR. In this study, all wild‐type (PC4^+/+^) and homozygous transgenic knock‐in mice (PC4^KI/KI^) were obtained from heterozygous mating.

### 4.4 Data and statistical analysis

Kaplan–Meier statistics were used to analyse survival in mice. All data are presented as means ± standard deviations. Statistical analyses were applied using the unpaired 2‐tailed Student's *t*‐test and one‐way (Bonferroni) analysis of variance with GraphPad Prism 7.04 statistical software. In all cases, *p *< 0.05 was considered statistically significant. In addition, asterisks denote statistical significance (ns, no significance; *p < 0.05).

Correlation analysis was carried out using the Pearson with GEPIA, a web server for cancer and normal gene expression profiling and interactive analyses (Tang et al., 2017).

Detailed information about resources and methods are available in the Supporting Information.

## CONFLICT OF INTERESTS

None declared.

## AUTHOR CONTRIBUTIONS

C.S. and L.C. involved in conceptualization and data interpretation. L.C., F.Y.L., J.W., Z.W. and Z.J. involved in experimental research and data analysis. Andreas Marx, M.L., Z.Y., S.H., Z.L. and Z.J. involved in intellectual input, scientific know‐how, reagents. L.C., C.Z., P.L., L.M., Q.G., Y.W. and Q.W. involved in writing–original draft. All authors involved in writing–review and editing. L.C., Z.Y., Y.W. and Z.W. revised the manuscript. C.S. involved in overall project supervision.

## Supporting information

Supplementary Material

Video S1

Video S2

## Data Availability

The data of co‐IP assays combined with mass spectrometry are available in Table S1. All other original data in this study are available from the authors on request.
