# Supplementary material for: Acceleration of ageing via disturbing mTOR‐regulated proteostasis by a new ageing‐associated gene PC4
Source: Aging Cell. 2021 May 6;20(6):e13370. doi: 10.1111/acel.13370 (PMC8208792; doi:10.1111/acel.13370)
Supplement: Supplementary file 1 — Supplementary Material [file ACEL-20-e13370-s002.docx]

**Supplementary figure legends**

**Supplementary figure 1** Correlation analysis of PC4 and aging- associated genes using RNA-seq datasets of in the skin (a) B kidney (b) and muscle (c) from GTEx.

**Supplementary figure 2** PC4 mRNA (a) and PC4 protein (b) level in heart, liver, spleen, skin, and kidney of adult PC4(+/+) and PC4(KI/KI) mouse. The data represent means ± SEM. (n=3, *P＜0.05, Student’s t-test). (c) Representative images of Lens opacity in 18-month-old PC4(+/+) and PC4(KI/KI) mouse.

**Supplementary figure 3** (a-b) The ubiquitinated protein level was detected through western blot in vivo (a) and in vitro (b). (c-e) Protein synthesis assessed by puromycin incorporation and detected by western blot in vivo (c) and in vitro (d and e). For (a and c) 18-month-old PC4(+/+) or PC4(KI/KI) mice were used. For (d and e) 2BS cells at 45 passage were used.

**Supplementary figure 4** (a) Protein synthesis assessed by puromycin incorporation and detected by western blot. Rapamycin (RAPA), a classical mTOR inhibitor. (b) Senescence-associated indicators including P21 and P16 in 18-month-old PC4(+/+) or PC4(KI/KI) mice after RAPA (75mg/kg) treatment for two weeks. n=3

**Figures**


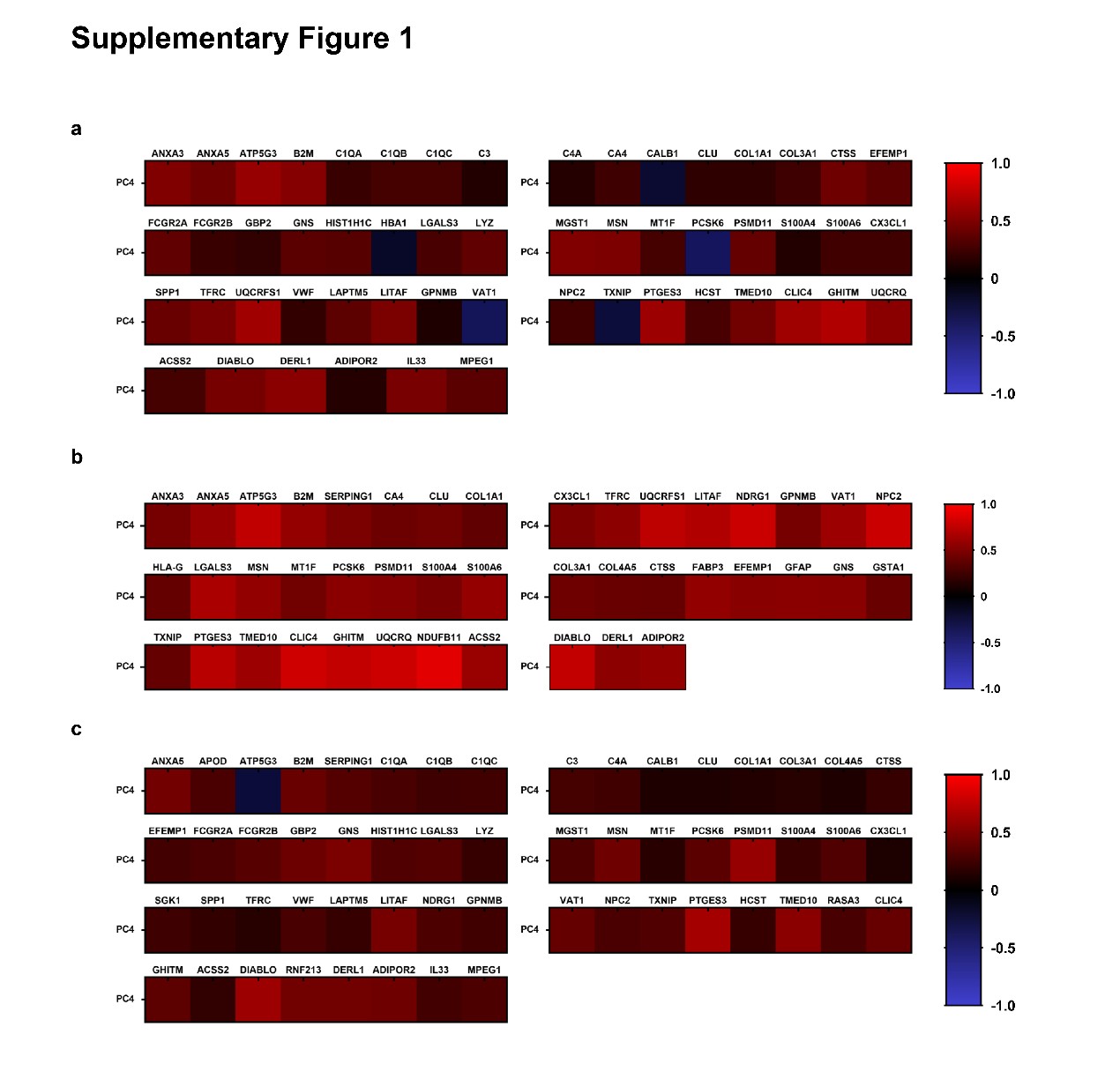


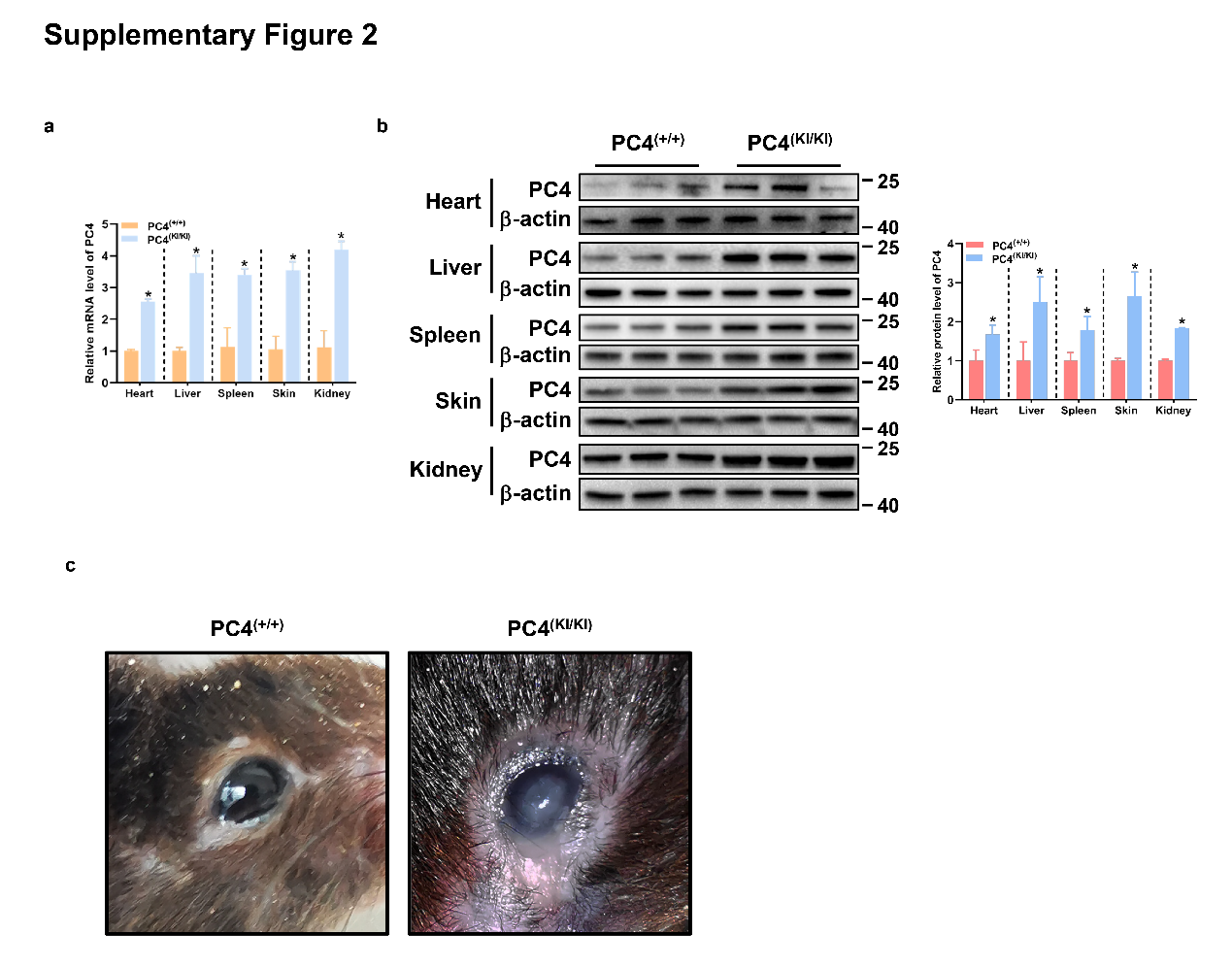


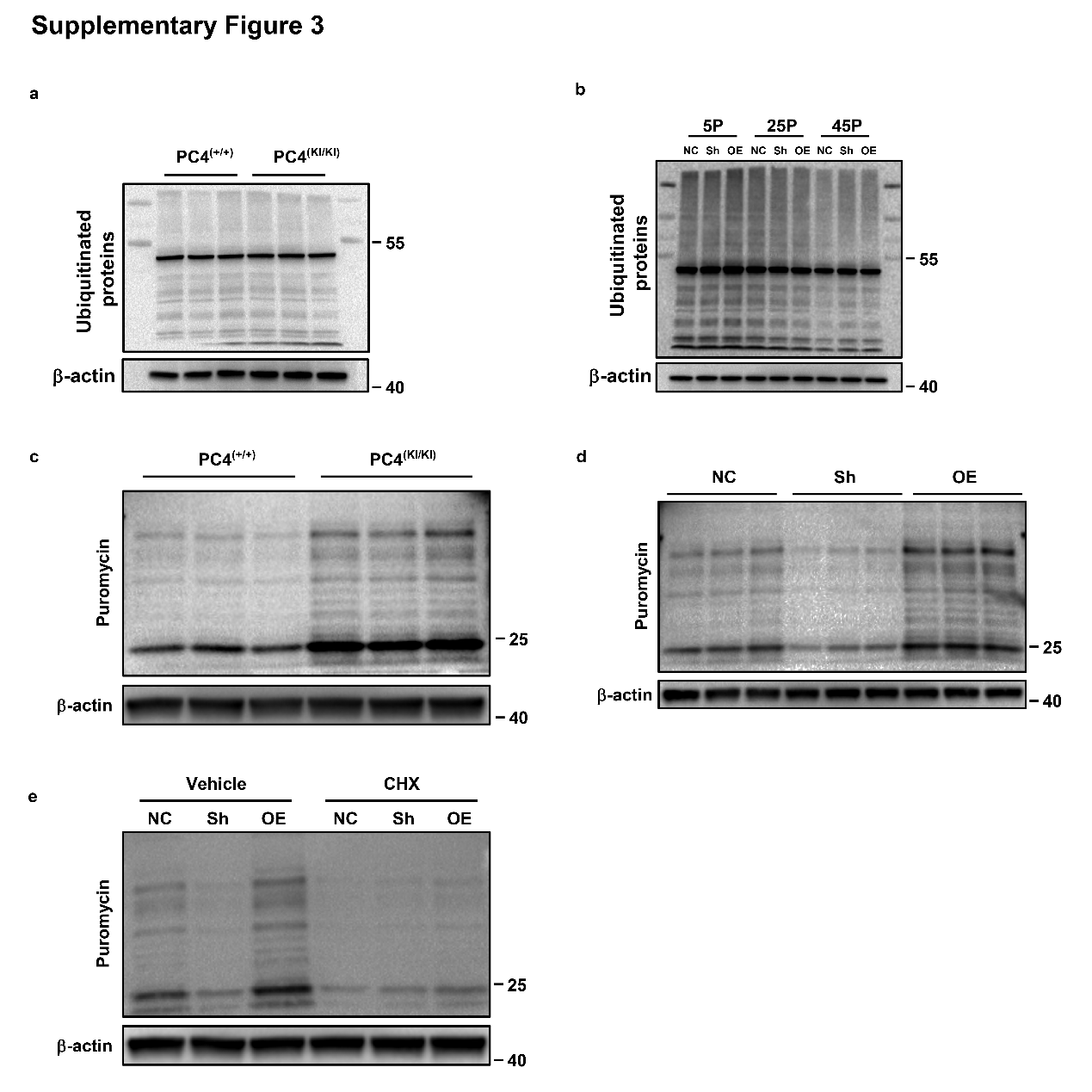


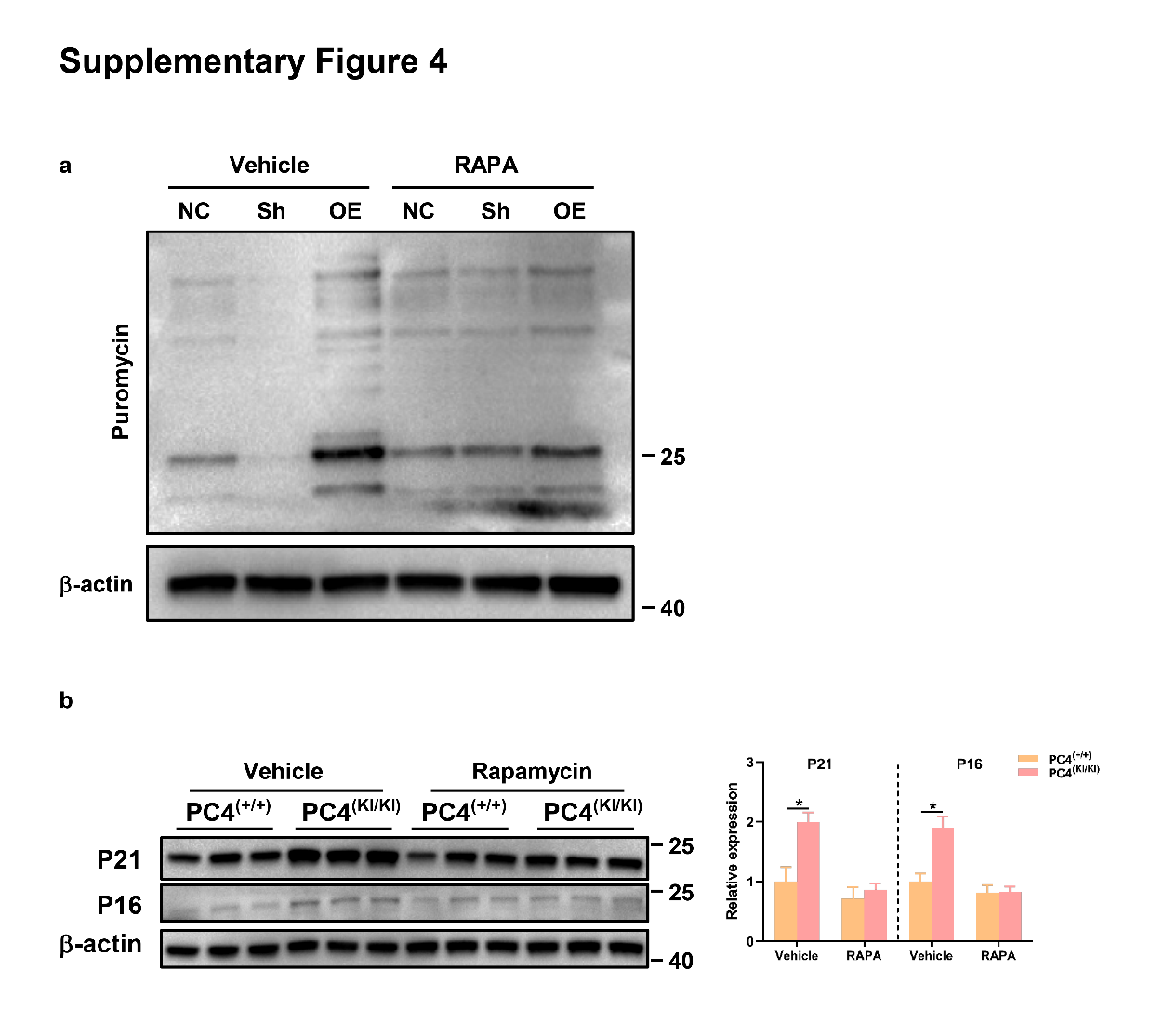


**Supplementary Table 1 Results of co-IP assays combined with mass spectrometry**

| Accession | Gene | ΣCoverage |
| --- | --- | --- |
| Q9NXV2 | KCTD5 | 63.25 |
| P60660 | MYL6 | 58.28 |
| P19105 | MYL12A | 57.31 |
| P16104 | H2AFX | 52.45 |
| P35580 | MYH10 | 48.43 |
| P53999 | SUB1 | 46.46 |
| P52907 | CAPZA1 | 39.86 |
| Q16643 | DBN1 | 37.90 |
| Q9Y5S9 | RBM8A | 35.63 |
| P23528 | CFL1 | 35.54 |
| P68431 | HIST1H3 | 35.29 |
| P50402 | EMD | 34.65 |
| Q7RTV0 | PHF5A | 34.55 |
| Q86WW8 | COA5 | 33.78 |
| Q86W42 | THOC6 | 32.84 |
| P62861 | FAU | 32.20 |
| Q9Y5V0 | ZNF706 | 30.26 |
| P36873 | PPP1CC | 28.79 |
| Q2TAY7 | SMU1 | 28.46 |
| P62633 | CNBP | 28.25 |
| P63244 | RACK1 | 27.13 |
| Q96HS1 | PGAM5 | 26.99 |
| P62273 | RPS29 | 26.79 |
| P38919 | EIF4A3 | 26.76 |
| Q15029 | EFTUD2 | 26.54 |
| P07437 | TUBB | 23.42 |
| O95232 | LUC7L3 | 22.92 |
| Q15388 | TOMM20 | 22.76 |
| P0DP24 | CALM2 | 22.15 |
| P68032 | ACTC1 | 22.02 |
| Q8N0T1 | RBIS | 22.00 |
| O75533 | SF3B1 | 20.78 |
| P61254 | RPL26 | 20.69 |
| O94832 | MYO1D | 20.68 |
| Q96FV9 | THOC1 | 20.55 |
| Q6P1L8 | MRPL14 | 20.00 |
| P10412 | HIST1H1 | 19.63 |
| O75643 | SNRNP20 | 19.62 |
| Q9NYL9 | TMOD3 | 19.60 |
| O75494 | SRSF10 | 19.47 |
| P41223 | BUD31 | 19.44 |
| O43809 | NUDT21 | 19.38 |
| P60866 | RPS20 | 19.33 |
| Q6P6C2 | ALKBH5 | 19.29 |
| Q15007 | WTAP | 19.19 |
| P67870 | CSNK2B | 19.07 |
| Q96DI7 | SNRNP40 | 19.05 |
| P08621 | SNRNP70 | 18.54 |
| P27797 | CALR | 17.51 |
| Q13247 | SRSF6 | 17.44 |
| P62857 | RPS28 | 17.39 |
| O43795 | MYO1B | 17.17 |
| P67936 | TPM4 | 16.94 |
| Q9P021 | CRIPT | 16.83 |
| Q9NQ29 | LUC7L | 16.71 |
| Q9NX58 | LYAR | 16.09 |
| Q6WCQ1 | MPRIP | 15.61 |
| P52732 | KIF11 | 15.34 |
| Q15004 | PCLAF | 15.32 |
| P62841 | RPS15 | 15.17 |
| P27694 | RPA1 | 15.10 |
| O43290 | SART1 | 15.00 |
| P42166 | TMPO | 14.99 |
| Q6P2Q9 | PRPF8 | 14.78 |
| P05141 | SLC25A5 | 14.77 |
| Q9UKV3 | ACIN1 | 14.39 |
| Q92743 | HTRA1 | 14.17 |
| P0CF74 | IGLC6 | 14.15 |
| P35244 | RPA3 | 14.05 |
| Q8NI27 | THOC2 | 13.68 |
| P11021 | HSPA5 | 13.46 |
| Q15393 | SF3B3 | 13.39 |
| Q9BQA1 | WDR77 | 13.16 |
| Q13595 | TRA2A | 13.12 |
| P26641 | EEF1G | 13.04 |
| Q96IZ0 | PAWR | 12.65 |
| P11142 | HSPA8 | 12.38 |
| P47756 | CAPZB | 12.27 |
| Q9UI30 | TRMT112 | 11.20 |
| Q69YN4 | VIRMA | 11.20 |
| Q9BRT6 | LLPH | 10.85 |
| P60842 | EIF4A1 | 10.84 |
| O15131 | KPNA5 | 10.82 |
| Q99549 | MPHOSPH | 10.81 |
| Q99417 | MYCBP | 10.68 |
| P06753 | TPM3 | 10.53 |
| O00422 | SAP18 | 10.46 |
| Q8WUA2 | PPIL4 | 10.37 |
| P46087 | NOP2 | 10.34 |
| P61326 | MAGOH | 10.27 |
| Q08170 | SRSF4 | 10.12 |
| O60828 | PQBP1 | 9.81 |
| Q9P2I0 | CPSF2 | 9.59 |
| P37108 | SRP14 | 9.56 |
| Q06830 | PRDX1 | 9.55 |
| P56182 | RRP1 | 9.54 |
| Q9Y3U8 | RPL36 | 9.52 |
| P52294 | KPNA1 | 9.48 |
| Q9BSD7 | NTPCR | 9.47 |
| Q15459 | SF3A1 | 9.46 |
| Q8NFW8 | CMAS | 9.45 |
| Q16630 | CPSF6 | 9.44 |
| Q9NV06 | DCAF13 | 9.44 |
| Q01804 | OTUD4 | 9.43 |
| A0A0C4DH31 | IGHV1-1 | 9.40 |
| P55769 | SNU13 | 9.38 |
| O75394 | MRPL33 | 9.23 |
| Q13769 | THOC5 | 9.22 |
| Q9Y237 | PIN4 | 9.16 |
| Q13435 | SF3B2 | 9.16 |
| P35610 | SOAT1 | 9.09 |
| P40938 | RFC3 | 8.99 |
| Q9H307 | PNN | 8.79 |
| O75152 | ZC3H11A | 8.64 |
| O14639 | ABLIM1 | 8.61 |
| Q5VTL8 | PRPF38B | 8.61 |
| O14744 | PRMT5 | 8.48 |
| O43172 | PRPF4 | 8.43 |
| Q8IYM9 | TRIM22 | 8.43 |
| Q8N5Z5 | KCTD17 | 8.41 |
| Q9UGN5 | PARP2 | 8.40 |
| P49756 | RBM25 | 8.19 |
| P18077 | RPL35A | 8.18 |
| Q9H7B2 | RPF2 | 8.17 |
| Q9NVS2 | MRPS18A | 8.16 |
| P15927 | RPA2 | 8.15 |
| O43716 | GATC | 8.09 |
| Q9Y3A2 | UTP11 | 7.91 |
| Q9GZV4 | EIF5A2 | 7.84 |
| P06733 | ENO1 | 7.83 |
| Q9BWF3 | RBM4 | 7.69 |
| P49207 | RPL34 | 7.69 |
| O14602 | EIF1AY | 7.64 |
| Q9ULV4 | CORO1C | 7.59 |
| Q15024 | EXOSC7 | 7.56 |
| Q96J01 | THOC3 | 7.41 |
| P62906 | RPL10A | 7.37 |
| Q9P0K7 | RAI14 | 7.24 |
| P35251 | RFC1 | 7.23 |
| P58743 | SLC26A5 | 7.12 |
| Q9UNZ5 | C19orf5 | 7.07 |
| Q9UHB6 | LIMA1 | 6.85 |
| Q99986 | VRK1 | 6.82 |
| P24534 | EEF1B2 | 6.67 |
| Q9NX63 | CHCHD3 | 6.61 |
| Q8TCX5 | RHPN1 | 6.57 |
| P09429 | HMGB1 | 6.51 |
| O15347 | HMGB3 | 6.50 |
| Q9UM13 | ANAPC10 | 6.49 |
| P45880 | VDAC2 | 6.46 |
| Q9NVI7 | ATAD3A | 6.31 |
| P31689 | DNAJA1 | 6.30 |
| Q13123 | IK | 6.28 |
| O15042 | U2SURP | 6.22 |
| P33993 | MCM7 | 5.98 |
| Q9UHY1 | NRBP1 | 5.98 |
| Q8IV63 | VRK3 | 5.91 |
| Q6I9Y2 | THOC7 | 5.88 |
| Q14247 | CTTN | 5.82 |
| P08727 | KRT19 | 5.75 |
| P55081 | MFAP1 | 5.69 |
| P84085 | ARF5 | 5.56 |
| Q86VM9 | ZC3H18 | 5.56 |
| Q7Z406 | MYH14 | 5.51 |
| Q9UQ35 | SRRM2 | 5.38 |
| P17026 | ZNF22 | 5.36 |
| O43660 | PLRG1 | 5.25 |
| P07305 | H1F0 | 5.15 |
| Q16610 | ECM1 | 4.81 |
| Q9Y4L1 | HYOU1 | 4.80 |
| Q96EY1 | DNAJA3 | 4.79 |
| O94906 | PRPF6 | 4.78 |
| P61964 | WDR5 | 4.49 |
| Q96CT7 | CCDC124 | 4.48 |
| Q13838 | DDX39B | 4.44 |
| P17029 | ZKSCAN1 | 4.44 |
| Q9H8G2 | CAAP1 | 4.43 |
| P14618 | PKM | 4.33 |
| Q13045 | FLII | 4.18 |
| Q09161 | NCBP1 | 4.18 |
| Q9P031 | CCDC59 | 4.15 |
| O95639 | CPSF4 | 4.09 |
| Q15428 | SF3A2 | 4.09 |
| Q75N03 | CBLL1 | 4.07 |
| Q9Y388 | RBMX2 | 4.04 |
| P38432 | COIL | 3.99 |
| Q9HBM6 | TAF9B | 3.98 |
| O60832 | DKC1 | 3.89 |
| Q9Y608 | LRRFIP2 | 3.88 |
| Q8NG66 | NEK11 | 3.88 |
| Q7Z7H8 | MRPL10 | 3.83 |
| Q15014 | MORF4L2 | 3.82 |
| Q05513 | PRKCZ | 3.72 |
| Q14978 | NOLC1 | 3.72 |
| P36542 | ATP5F1C | 3.69 |
| Q6PJT7 | ZC3H14 | 3.67 |
| Q01130 | SRSF2 | 3.62 |
| Q16891 | IMMT | 3.56 |
| Q96JN0 | LCOR | 3.46 |
| Q13427 | PPIG | 3.45 |
| P19784 | CSNK2A2 | 3.43 |
| O43395 | PRPF3 | 3.37 |
| Q8N9M1 | C19orf4 | 3.32 |
| Q00325 | SLC25A3 | 3.31 |
| Q15427 | SF3B4 | 3.30 |
| Q15050 | RRS1 | 3.29 |
| P27348 | YWHAQ | 3.27 |
| Q9Y512 | SAMM50 | 3.20 |
| Q9NW13 | RBM28 | 3.16 |
| Q9UBB9 | TFIP11 | 3.11 |
| Q9UPT8 | ZC3H4 | 3.07 |
| O60870 | KIN | 3.05 |
| Q02742 | GCNT1 | 3.04 |
| Q9NNW5 | WDR6 | 3.03 |
| Q9NQ55 | PPAN | 2.96 |
| Q9HAZ1 | CLK4 | 2.91 |
| O60306 | AQR | 2.83 |
| Q8IWX8 | CHERP | 2.73 |
| P25705 | ATP5F1A | 2.71 |
| Q96EU6 | RRP36 | 2.70 |
| P49916 | LIG3 | 2.68 |
| Q9Y250 | LZTS1 | 2.68 |
| Q53GS9 | USP39 | 2.65 |
| P02749 | APOH | 2.61 |
| Q96MM3 | ZFP42 | 2.58 |
| Q5M9Q1 | NKAPL | 2.49 |
| Q7Z6E9 | RBBP6 | 2.46 |
| Q9H7N4 | SCAF1 | 2.44 |
| Q6GYQ0 | RALGAPA | 2.41 |
| Q9UPU9 | SAMD4A | 2.37 |
| Q92620 | DHX38 | 2.36 |
| O95819 | MAP4K4 | 2.34 |
| Q9NWF9 | RNF216 | 2.31 |
| P05156 | CFI | 2.23 |
| Q9HCG8 | CWC22 | 2.20 |
| Q13523 | PRPF4B | 2.18 |
| P23588 | EIF4B | 2.13 |
| Q9P2D0 | IBTK | 2.07 |
| Q8WUM0 | NUP133 | 1.99 |
| Q8N9T8 | KRI1 | 1.99 |
| P07996 | THBS1 | 1.97 |
| O75815 | BCAR3 | 1.94 |
| Q9Y5B9 | SUPT16H | 1.91 |
| Q5VWN6 | TASOR2 | 1.89 |
| Q02040 | AKAP17A | 1.87 |
| Q96L12 | CALR3 | 1.82 |
| O14647 | CHD2 | 1.81 |
| P02751 | FN1 | 1.80 |
| P10909 | CLU | 1.78 |
| Q9NZM6 | PKD2L2 | 1.76 |
| O14654 | IRS4 | 1.75 |
| O14646 | CHD1 | 1.70 |
| Q96T83 | SLC9A7 | 1.66 |
| Q96GQ7 | DDX27 | 1.63 |
| Q8NHP8 | PLBD2 | 1.53 |
| Q96MU7 | YTHDC1 | 1.51 |
| Q9Y3T9 | NOC2L | 1.47 |
| Q92802 | N4BP2L2 | 1.37 |
| Q9BUQ8 | DDX23 | 1.34 |
| Q9NWH9 | SLTM | 1.26 |
| Q8IYB3 | SRRM1 | 1.22 |
| Q96EY7 | PTCD3 | 1.16 |
| Q14157 | UBAP2L | 1.01 |
| Q14643 | ITPR1 | 0.98 |
| P38398 | BRCA1 | 0.97 |
| Q01082 | SPTBN1 | 0.93 |
| O75923 | DYSF | 0.82 |
| P02671 | FGA | 0.81 |
| Q92698 | RAD54L | 0.80 |
| Q86UP3 | ZFHX4 | 0.76 |
| Q9Y239 | NOD1 | 0.73 |
| Q14683 | SMC1A | 0.73 |
| Q5T9S5 | CCDC18 | 0.62 |
| P78527 | PRKDC | 0.61 |
| P46013 | MKI67 | 0.58 |
| Q15075 | EEA1 | 0.50 |
| Q5THJ4 | VPS13D | 0.48 |
| P15924 | DSP | 0.35 |
| Q8WZ42 | TTN | 0.03 |

**Footnotes:**

PC4-binding proteins were identified through Co-IP assays combined with mass spectrometry.

**Supplementary Table 2 Functional Categories (UP KEYWORDS) analysis results**

| **Category** | **Term** | **Count** | **%** | **PValue** |
| --- | --- | --- | --- | --- |
| UP_KEYWORDS | Protein biosynthesis | 8 | 0.027449904 | 9.25187E-05 |
| UP_KEYWORDS | Actin-binding | 6 | 0.020587428 | 0.000259625 |
| UP_KEYWORDS | Coiled coil | 39 | 0.133818282 | 0.000349952 |
| UP_KEYWORDS | Ribonucleoprotein | 9 | 0.030881142 | 0.000592583 |
| UP_KEYWORDS | Motor protein | 5 | 0.01715619 | 0.001526059 |
| UP_KEYWORDS | Myosin | 4 | 0.013724952 | 0.003231532 |
| UP_KEYWORDS | Ribosomal protein | 6 | 0.020587428 | 0.008349135 |
| UP_KEYWORDS | Initiation factor | 4 | 0.013724952 | 0.01698438 |
| UP_KEYWORDS | ATP-binding | 13 | 0.044606094 | 0.031053526 |
| UP_KEYWORDS | Elongation factor | 3 | 0.010293714 | 0.035639097 |
| UP_KEYWORDS | Nucleotide-binding | 15 | 0.05146857 | 0.037256574 |
| UP_KEYWORDS | Helicase | 3 | 0.010293714 | 0.09100843 |

**Footnotes:**

Functional Categories (UP KEYWORDS) analysis results of proteins identified to might interact with PC4 though co-IP assays combined with mass spectrometry (MS).

**Methods and Resources**

**Supplementary Table 3. Agents**

| **Antibodies** | **Source** | **Cat#** |
| --- | --- | --- |
| anti-rabbit PC4 | Novus Biologicals | NB10059774, RRID: N/A |
| PE/Cyanine7 anti-mouse CD3 Antibody | Biolegend | 100219, RRID: AB_1732068 |
| BV510 anti-mouse CD4 Antibody | Biolegend | 100449, RRID: AB_2564587 |
| PE anti-mouse CD62L Antibody | Biolegend | 104407 RRID: AB_313094 |
| PerCP anti-mouse/human CD44 Antibody | Biolegend | 103036, RRID: AB_10645506 |
| APC anti-mouse CD8a Antibody | Biolegend | 155006, RRID: AB_2750213 |
| Anti-mouse Pol II | Sigma | 05-623, RRID: AB_2257634 |
| Anti-mouse TFIIB | Santa Cruz Biotechnology | SC271736, RRID: AB_2633087 |
| anti-mouse Acetylated Lysine | Thermo Scientific | MA1-2021, RRID: AB_881819 |
| anti-rabbit Puromycin | Thermo Scientific | 702389, RRID: AB_10861551 |
| anti-rabbit P16 | Abcam | ab54210, RRID: AB_881819 |
| anti-rabbit p21 | Abcam | ab109199, RRID:AB_10861551 |
| anti-rabbit H3K9ac | Abcam | ab4441, RRID: AB_2118292 |
| anti-rabbit LC3B | Bimake | A5202, RRID: N/A |
| anti-rabbit γ-H2AX | Cell Signaling Technologies | 9718S, RRID: AB_2118009 |
| Anti-rabbit IgG, HRP-linked | Cell Signaling Technologies | 7074S, RRID: AB_2099233 |
| Anti-mouse IgG, HRP-linked | Cell Signaling Technologies | 7076S, RRID: AB_330924 |
| anti-rabbit Rheb | Cell Signaling Technologies | 13879S, RRID: AB_2721022 |
| anti-rabbit mTOR | Cell Signaling Technologies | 2983S, RRID: AB_2105622 |
| anti-rabbit Phospho-mTOR | Cell Signaling Technologies | 5536S, RRID: AB_10691552 |
| anti-rabbit Phospho-S6 Ribosomal Protein | Cell Signaling Technologies | 2708S, RRID: AB_390722 |
| anti-rabbit S6 Ribosomal Protein | Cell Signaling Technologies | 2217S, RRID: AB_331355 |
| anti-rabbit p70 S6 Kinase | Cell Signaling Technologies | 2708S, RRID: AB_390722 |
| anti-rabbit Phospho-p70 S6 Kinase | Cell Signaling Technologies | 9234S, RRID: AB_2269803 |
| Anti-mouse Ubiquitin | Cell Signaling Technologies | 3936S, RRID: AB_331292 |
| anti-mouse β-Actin | Cell Signaling Technologies | 3700S, RRID: AB_2242334 |
| anti-rabbit SAP18 Antibody | PROTEINTECH | 13841-1-AP, RRID: AB_2301353 |
| **Chemicals** | **Source** | **Cat#** |
| Rapamycin | Selleckchem | S1039 |
| Puromycin | Selleckchem | S7417 |
| Cyclohexanecarboxamide | Selleckchem | S6122 |
| ITSA-1 | Selleckchem | S8323 |
| **Assay Kits** | **Source** | **Cat#** |
| Cell Counting Kit-8 | Dojindo Molecular Technologies | ck04 |
| Crystal violet | Beyotime | C0121 |
| Senescence Detection Kit | Dojindo Molecular Technologies | SG03 |
| H&E Staining Kit | Abcam | ab245880 |
| DAB substrate kit | Abcam | ab64238 |
| Protease Inhibitor Cocktail | Roche | 5892791001 |
| Revert Aid First Strand cDNA Synthesis Kit | Thermo Scientific | K1622 |
| BCA Protein Assay Kit | Thermo Scientific | A53225 |
| Proteasome 20S Activity Assay Kit | Sigma | MAK172 |
| PROTEOSTAT Protein aggregation assay | Enzo Life Science | ENZ-51023 |
| Simple ChIP Enzymatic Chromatin IP Kit | Cell Signaling Technology | 9003S |

**Supplementary Table 4. Sequences of the primers used for qRT-PCR**

| **Gene** | **Forward sequences** | **Reverse sequences** |
| --- | --- | --- |
| Mouse PC4/SUB1 | 5-ATGTCAGTGTTCGGGACTTCA-3 | 5-ACAGCTTTCTTACTGCGTCATC-3 |
| Mouse GAPDH | 5-GGCATCCTGGGCTACACT-3 | 5-CCACCACCCTGTTGCTGT-3 |
| Human PC4/SUB1 | 5-GGTGAGACTTCGAGAGCCCT-3 | 5-GGTGAGACTTCGAGAGCCCT-3 |
| Human IL1α | 5-AATGACGCCCTCAATCAAAG-3 | 5-TGGGTATCTCAGGCATCTCC-3 |
| Human IL1β | 5-ATGATGGCTTATTACAGTGGCAA-3 | 5-GTCGGAGATTCGTAGCTGGA-3 |
| Human IL6 | 5-ACTCACCTCTTCAGAACGAATTG-3 | 5-CCATCTTTGGAAGGTTCAGGTTG-3 |
| Human IL8 | 5-ACTGAGAGTGATTGAGAGTGGAC-3 | 5-AACCCTCTGCACCCAGTTTTC-3 |
| Human MMP3 | 5-AGTCTTCCAATCCTACTGTTGCT-3 | 5-TCCCCGTCACCTCCAATCC-3 |
| Human MMP12 | 5-GATCCAAAGGCCGTAATGTTCC-3 | 5-TGAATGCCACGTATGTCATCAG-3 |
| Human PAI1 | 5-TGGGTGAAGACACACACAAAAGG-3 | 5-GTGCTGGAGTCGGGGAAGG-3 |
| Human GAPDH | 5-GGCATCCTGGGCTACACT-3 | 5-CCACCACCCTGTTGCTGT-3 |

**Supplementary Table 5. Sequences of the primers used for qRT-PCR (Chip)**

| **Gene** | **Forward sequences** | **Reverse sequences** |
| --- | --- | --- |
| Rheb | 5-GGTCTGTGGGAAAGTCCTC-3 | 5-TGTTCTCTATGGTTGGATCGT-3 |
| mTOR | 5-CACAAGGAGATCCGCATGGA-3 | 5-GCGGATATCAGGGTCAGGAT-3 |
| p70S6K | 5-CACCTGTCAGCCCAGTCAAA-3 | 5- CCGCTCACTGTCACATCCAT-3 |
| RAGA | 5-TTCCATCTTAGGGCTCACGC-3 | 5-TTCCATCTTAGGGCTCACGC-3 |
| RAGB | 5-TTCCATCTTAGGGCTCACGC-3 | 5-TTCCATCTTAGGGCTCACGC-3 |
| RAGC | 5-TTCCATCTTAGGGCTCACGC-3 | 5-TTCCATCTTAGGGCTCACGC-3 |
| RAGD | 5-TTCCATCTTAGGGCTCACGC-3 | 5-TTCCATCTTAGGGCTCACGC-3 |
| TSC2 | 5-TTCCATCTTAGGGCTCACGC-3 | 5-TTCCATCTTAGGGCTCACGC-3 |

**Supplementary Table 6. Sequences of the PC4 shRNA lentivirus vector**

| **Gene** | **Forward sequences** | **Reverse sequences** |
| --- | --- | --- |
| Negative control | 5-UUCUCCGAACGUGUCACGUTT-3 | 5-ACGUGACACGUUCGGAGAATT-3 |
| Knockdown | 5-ACAGAGCAGCAGCAGCAGATT-3 | 5-UCUGCUGCUGCUGCUCUGUTT-3 |

**Supplementary methods**

**Cell culture**

The human fetal lung diploid fibroblast 2BS (RRID: CVCL_BT02) cell line was purchased from the National Institute of Biological Products (Beijing, China) and maintained in a humidified incubator with 5% CO2 at 37 °C, cultured in the recommended medium and confirmed to be free of Mycoplasma.

**GEO microarray analyses**

Data set GSE9103 including 40 healthy participants’ vastus lateralis was collected through NCBI GEO searching. The young participants were at the age of 18–30 years and the old participants were at the age of 59–76 years.

**Correlation analysis between PC4 and ageing-related genes**

73 ageing-related genes which commonly altered during ageing were collected from Garage, and correlation analysis was performed with PC4.

**Micro-CT**

Mice were scanned under anaesthesia using a Micro-CT (GE Healthcare, Ontario, Canada) at the 20-μm resolution, 2000 s exposure time, 0.5 angles of increment, and 80 kV and 450 μA. Four trained technicians were invited to blindly determine the mean CT value and standard deviation for every region of interest using MicroView 2.1 software.

**Transfection and generation of stable cell lines**

2BS cells (1 × 10^6^ to 2 × 10^6^ cells) were seeded in a 6-well plate before infected at a multiplicity of infection (MOI) of 20 with control, PC4-knockdown or PC4-overexpression virus (GeneChem; Shanghai, China) and incubated for 12 h at 37 °C, then washed with PBS and cultured in complete medium for 72 h, followed by selected GFP positive cells by flow sorting to obtain the stable cell line.

**Cell** **proliferation**

The cell proliferation was assessed using the Cell Counting Kit-8 (Dojindo Lab, Tokyo, Japan). Briefly, 5000 cells per well were seeded in 96-well plates and cultured for the indicated time. Next, 10 µL of CCK-8 solution was added to each well and incubated for 2 h at 37 ℃, then the absorbance was measured at 450 nm using a spectrophotometer.

**EdU incorporation analysis**

EdU incorporation was evaluated by the average fluorescence intensity using Click-It EdU Flow Cytometry Imaging Kit according to the manufacturers’ protocol.

**Apoptosis assay**

Briefly, cells were washed twice in PBS and then harvested and then stained using the Dead Cell Apoptosis Kit at room temperature in the dark for 15 min, and finally subjected to flow cytometry (Accuri C6, BD Biosciences). The apoptosis cells were defined as those which were positive for Annexin V.

**Colony formation**

1000 cells per well were seeded in 6-well plates, and the fresh medium was changed every 3 days until colonies were clearly visible (about 2 weeks). Then the colonies were washed with PBS, fixed, stained with Crystal Violet Staining Solution for 15 minutes, and then counted (at least 50 cells for a colony).

**Senescence-associated beta-galactosidase (SA-β-gal) analysis**

Cells were seeded in 6-well plates and cultured at 37 ℃ overnight. Then removed the medium and washed twice with Hanks' Balanced Salt Solution (HBSS) and stained the cells according to the manufacturer’s protocol. After washed, cells were isolated with trypsin and detected through flow cytometry. The mean fluorescence intensity was used for analysis.

**T lymphocyte subsets**

The blood was collected using a Heparin sodium treated tube from the heart under anaesthesia, then erythrocyte was lysed. After washing twice, samples were incubated with antibodies (CD3, CD4, CD44 and CD62L) for 30 min at room temperature. Then samples were analyzed using a fluorescence-activated cell sorter (FACS)verse (BD Biosciences, San Jose, CA) flow cytometer within 30 minutes.

**Hematologic parameter test**

20 μL of peripheral blood from the tail veins of the mice was added to 180 μL 1% ethylene diamine tetraacetic acid solution, and then detected automatically using a Sysmex XT-2000iV haematology analyzer (Kobe, Japan).

**Histology**

Mice were sacrificed by euthanasia. The tissues of mice were collected and fixed in 4% formaldehyde, dehydrated, embedded in paraffin, cut into 5 μm-thick sections, deparaffinized and rehydrated. For H&E, sections were stained according to the manufacturer’s protocol. For IHC, sections were antigen retrieved using 1 M sodium citrate buffer (pH 6.5). Endogenous peroxidase was blocked using 3% H2O2 for 10 min. Furthermore, sections were blocked for 1 h at room temperature and then incubated with primary antibodies (1:200) at 4 °C overnight, followed by incubation with the secondary antibody for 1 h. Then nuclei were stained with hematoxylin.

The thickness of the dermal and adipose layers of skin was objectively analysed and quantified through photographs of H&E stained sections from at least five mice per group using ImageJ 1.37.

**Total protein analysis**

10^5^ cells per group were collected. The total RNA was extracted using PureLink® RNA Mini Kit (Thermo Scientific, Waltham, MA, USA). Then the total RNA concentration was assessed using NanoDrop 2000 spectrophotometer (Thermo Scientific). For the total protein, cells were lysed in RIPA buffer containing protease inhibitors and phosphatase inhibitors on ice. After centrifuged, the total protein concentration was assessed using a BCA kit.

**Protein aggregation**

Protein aggregation was measured using the ProteoStat^®^ Protein aggregation assay according to the manufacturer’s protocol. Briefly, cells were lysed with lysis buffer (HEPES 50 mM, NaCl 150 mM, EDTA 5 mM, DTT 2 mM), and the concentrations were detected using a BCA kit. Then the lysate containing equal amounts of proteins was added to a black 96-well microplate and incubated with loading solution for 15 min in the dark. Finally, the fluorescence was detected using a fluorescence microplate reader (Ex: 544 nm, Em: 646 nm).

**Protein synthesis analysis**

SUnSET assays and Reporter gene assays were used to assess protein synthesis.

For SUnSET assays, cells were incubated with 1 μM puromycin for 30 min before collecting. In vivo, the mice were injected with puromycin (40 nmol/g; i.p.) for 30 mins before euthanasia with CO2 gas. Then the cells or tissues were performed according to the protocol of western blotting using a specific anti-Puromycin antibody.

For reporter gene assays, Luciferase reporter assays were performed using the Dual Luciferase Reporter Assay System from Promega according to manufacturer instructions. Briefly, every sample was divided into two groups, one of which was used to detect the mRNA level of GAPDH and Renilla luciferase gene through qPCR, and the other was lysed and detect the luciferase activity. The relative luciferase units were normalized against the mRNA levels of Renilla luciferase gene quantified by GAPDH.

**Detection of proteasomal activity**

Proteasome activity was detected using a Proteasome 20S Activity Assay Kit according to the manufacturer’s protocol. The relative proteasomal activity was calculated according to the fluorescence value of LLVY-AMC cleavage.

**Quantitative real-time PCR analysis**

Total RNA was extracted using PureLink® RNA Mini Kit (Thermo Fisher Scientific). cDNA synthesis was performed following the manufacturer’s protocol with RevertAid First Strand cDNA Synthesis Kit (Thermo Fisher Scientific). Real-time PCR was performed using an SYBR Green qPCR master mix (Takara). The primers are listed in Supplementary Table 2. In addition, all data were normalised to the control using GAPDH as the internal control by the ΔCT method.

**Western blotting**

Cells or tissues were lysed in RIPA buffer containing protease inhibitors and phosphatase inhibitors on ice for 30 min, and then centrifuged at 16 000 × g for 15 min at 4 °C. The concentrations were detected using a BCA kit (Thermo Fisher Scientific). The sample was heated with loading buffer (Beyotime) to 100 °C for 5 min, then subjected to SDS-PAGE, followed by transfer to a PVDF membrane (Merck Millipore, Darmstadt, Germany). The membranes were blocked for 1 hour at room temperature, and then immunoblotted with primary antibodies (1:1000) overnight at 4 °C and washed with Tris-buffered saline with Tween (TBST) buffer three times (5 minutes once), followed by incubation with secondary antibodies for 1 hour. After washing three times, the intensity of bands was determined using an enhanced chemiluminescence detection system (Bio-Rad Laboratories) by an ECL kit (Thermo Scientific, Waltham, USA). β-Actin was used as the loading control. In addition, Lysates were sampled from three samples per group.

Western blotting quantification was evaluated using Image Lab 3.0 software (Bio-rad).

**Immunoprecipitation**

Cell lysate containing 500 µg protein was incubated with indicated antibody or IgG control with rotation at 4 ℃ overnight. Then the immunoprecipitated complexes were combined with Protein A/G magnetic Beads and separated using eluent, finally, the samples were analyzed through Western Blotting.

**Acetylation detection of PC4**

Each sample was lysed with 200 μL whole cell lysate, then 20 μL of the lysate was used to perform the Western blotting procedure. The remaining lysate was used to perform the Immunoprecipitation procedure using anti-acetylated lysine antibody, then samples were detected through Western blotting using an anti-PC4 antibody.

**Chromatin Immunoprecipitation (ChIP)**

SimpleChIP® Enzymatic Chromatin IP Kit was used to perform chip as manufacturer’s instructions. In brief, 4 X 10^6^ cells were prepared and incubated for 10 min with 1% formaldehyde to crosslink proteins to DNA and then quenched with 0.125 M glycine. The cells were washed with ice-cold PBS, enzymatically digested, and incubated with the indicated antibody. The immunoprecipitated complexes were pelleted with magnetic beads, then decrosslinked. The total DNA was purified and analysed by quantitative real-time PCR. The primers are listed in Supplementary Table 3 and normal rabbit IgG was used as a control to normalize.
